# Combined femoral and sciatic nerve blocks for lower limb anaesthesia in anticoagulated patients with severe cardiac valvular lesions

**DOI:** 10.4103/0019-5049.65372

**Published:** 2010

**Authors:** Thrivikrama Padur Tantry, Dinesh Kadam, Pramal Shetty, Sanath Bhandary

**Affiliations:** Department of Anaesthesiology, A J Institute of Medical Sciences, Kuntikana, Mangalore, India; 1 Department of Plastic & Reconstructive Surgery, A J Institute of Medical Sciences, Kuntikana, Mangalore, India

**Keywords:** Anticoagulation, lower limb anaesthesia, peripheral nerve blocks

## Abstract

Peripheral nerve block (PNB) in anticoagulated patients is controversial and guidelines are not defined. We report two patients with severe cardiac valvular lesions, who underwent emergency surgeries for lower limb. Both the patients were on anticoagulants, warfarin and heparin in one and aspirin and clopidogrel in the other, with abnormal coagulation profile in the former. Combined femoral and sciatic nerve blocks were used as a sole anaesthetic technique. Postoperatively, the patients were evaluated for bleeding complications at the injection site using high-frequency ultrasound probe. Both had uneventful surgery and recovery. A close postoperative monitoring following PNBs in anticoagulated patients is necessary.

## INTRODUCTION

Patients with severe valvular cardiac lesions pose anaesthetic challenge due to fixed cardiac outputs.[1] Both general anaesthesia (GA) and regional anaesthesia with central neuraxial blockade (CNB) carry potential risks. This is further complicated as these patients are generally on anticoagulants where any type of regional anaesthesia is contraindicated. Often these patients are prone for peripheral thromboembolic events necessitating emergency surgery for limb salvage. No specific anaesthetic guidelines are available for the management in such situations. The use of peripheral nerve blocks in cardiac patients who are on anticoagulation therapy is also controversial. We report two patients with incapacitating cardiac illness, who were on anticoagulants and successfully underwent emergency surgery of the lower limb under peripheral nerve block (PNB). Combined femoral and sciatic nerve blocks were used in both the patients as a sole anaesthetic technique. In life-threatening emergencies, PNBs are worthwhile to consider when administered with extreme care and monitored closely to detect haematoma postoperatively.

## CASE REPORTS

### Case 1

A 42-year-old lady with 56-kg body weight, a known patient of severe rhumatic valvular heart disease with pulmonary hypertension, was referred to our hospital with severe acute pain in left lower limb, which developed 16 hours before, due to acute femoral thromboembolic occlusion. She had exertional dyspnoea NYHA class II with palpitation and was receiving warfarin, diuretics, bronchodilators and digoxin. Intravenous heparin 5000 IU was given at a referring hospital 2 hours prior to our procedure. Clinical diagnosis of acute femoral artery thrombo-embolic occlusion with scanty distal flow was confirmed with color doppler. Investigation showed haemoglobin of 16.5 g% with total leukocyte count of 11,400/ml, serum creatinine 1.2 mg/dl, International Normalised Ratio 1.61, Bleeding Time and Clotting Time 2.3 and 5.0 minutes, respectively, prolonged activated Partial Thromboplastin Time beyond measurement and serum K^+^ 5.2 meq/l. Electrocardiogram showed atrial fibrillation (AF) with ST-T changes, echocardiogram revealed AF without atrial thrombus, severe mitral stenosis (valve area 0.9 cm^2^), grade II mitral regurgitation, severe aortic stenosis (peak pressure gradient/mean pressure gradient 75/51 mm Hg), severe pulmonary hypertension, severe tricuspid regurgitation with left ventricular hypertrophy. The patient also had poor pulmonary function with room air saturation of 91%.

The patient was taken for emergency thrombo-embolectomy. A combined femoral sciatic nerve block was planned. In supine position, a 22-G, 2.5-inch nerve locator (INMED, locator, HN) needle was used for femoral block; posterior branch of femoral nerve was located (by patellar ascension, dancing patella). A mixture of 20 ml local anaesthetic (15 ml of 0.5% bupivacaine + 5 ml of 2% lignocaine) was injected into the femoral sheath with a firm distal digital pressure. Fifteen minutes later, the patient had adequate pain relief. The patient repositioned laterally to perform sciatic block with the classic approach of Labat. Thirty milliliters of 0.25% bupivacaine was injected into the sheath of sciatic nerve using 22-G 10-cm needle (INMED, locator, HN) to identify the nerve eliciting motor responses of plantar flexion.

**Figure 1:**
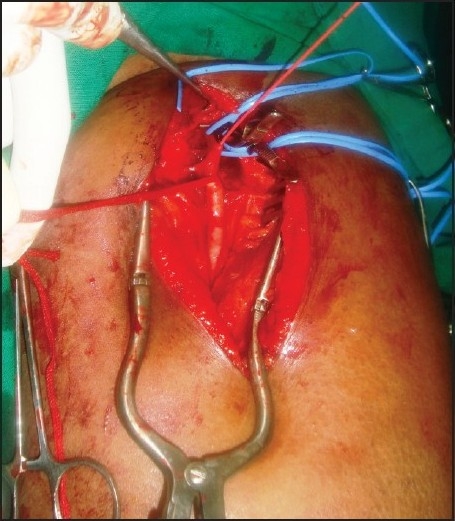
Femoral embolectomy

**Figure 2:**
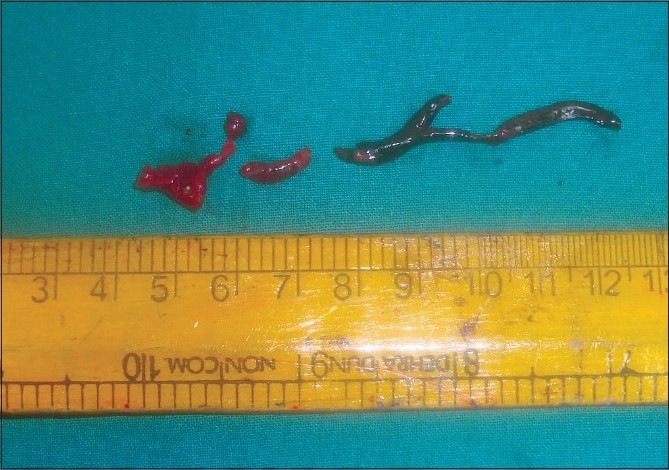
Embolus

After 20 minutes of sciatic block, the patient was totally relieved of pain and surgical embolectomy [Figures 1 and 2] was performed. Arterial and limb venous blood gas analysis was done prior to and following embolectomy. Total blood loss was 150 ml. Intravenous 2500 units of heparin was administered. Patient was monitored in postoperative ICU. An ultrasound (10 MHz, higher linear frequency probe, Acuson150, Seimens, California, USA) guided evaluation was carried out to rule out haematoma formation and compression on nerves at both sites, twice, till the complete motor and sensory recovery, which was after 7.5 hours. The patient had uneventful recovery and she was discharged on the eighth day.

### Case 2

**Figure 3:**
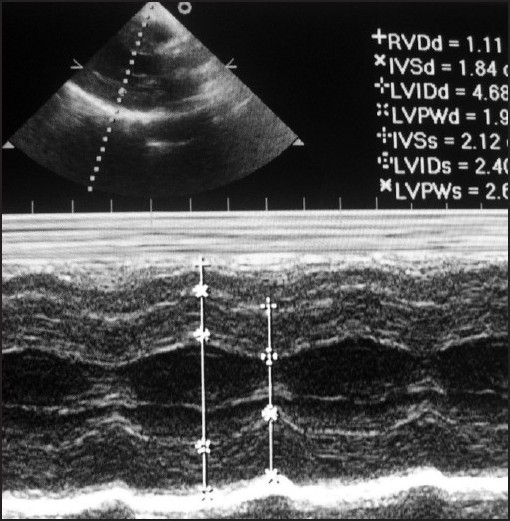
Echocardiogram of patient with severe aortic stenosis

A 73-year-old 61-kg male hypertensive and diabetic with peripheral vascular disease (PVD) patient was posted for emergency lower limb below knee amputation for gangrene of foot. Patient was a known alcoholic and smoker (30 pack years), had symptoms of syncopal attacks, dyspnoea and easy fatigability. Patient was on tab. atenolol 25 mg OD, tab. aspirin 75 mg OD, tab. clopidogrel 75 mg OD, tab. pentoxyfilline 400 mg TID for the past 4 months. Investigation showed serum creatinine levels of 1.5 mg/dl, blood sugar 212 mg/dl, Bleeding Time and Clotting Time 4 and 6.5 minutes, respectively. Electrocardiogram showed hypertrophied left ventricle and T inversions. Echocardiogram [Figure 3] revealed severe aortic stenosis with a mean gradient of 112 mm Hg with left ventricular hypertrophy.

Nerve blocks were performed with similar approaches as in case 1. Femoral nerve block was performed with mixture of 10 ml of 0.5% bupivacaine, 4 ml of isotonic saline 0.9% and 6 ml of 2% lignocaine (total volume 20 ml). Sciatic block was performed with 25 ml of 0.5% bupivacaine. Adequate block was achieved in 30 minutes. Surgery was completed uneventfully. Complete sensory and motor recovery was seen after 9 hours. An ultrasound-guided evaluation was carried out in the postoperative period (at 2^nd^ and 10^th^ hours) to rule out haematoma at the sites of injections.

## DISCUSSION

Severe mitral and aortic stenosis with severe pulmonary hypertension presents high risk group for CNB or GA.[1] Any hypoxia, hypercarbia, acidosis, tachycardia (due to pain, sympathetic stimulation or GA), etc., can worsen pulmonary edema or ischaemia.

Most commonly used anaesthetic technique for lower limb procedure is CNB, either spinal or epidural anaesthesia. However, its use is limited in compromised cardiac status due to frequent hypotension caused by sympathetic blockade.[2] Additionally, if these patients are on cardio-prophylactic anticoagulants, administering CNB is contraindicated leaving very little option when they need emergency surgery.

Both the patients who needed emergency lower limb surgery had fixed cardiac output conditions of severe form. Their condition was potentially life threatening. Administering GA or CNB was not without risks. Considering the above factors and available techniques, the combined femoral and sciatic nerve blocks[3] for lower limb anaesthesia was planned.

Administering heparin in vascular surgeries peroperatively alters coagulation. Risks of bleeding complications are well known in continuous peripheral nerve catheters. Guidelines for administration of regional PNBs in patients with anticoagulation therapy are not specifically defined.

Several cases of serious bleeding complications associated with PNB and following psoas compartment (lumbar plexus) block have been reported in patients with perioperative heparin, Low Molecular Weight Heparin (LMWH) or warfarin.[4,5] Bleeding-related complication like large retroperitoneal haematomas[6] was seen more frequently than neural deficits. Delayed (>9 hours) haematoma formation was reported even when antithrombotics were stopped 3 days prior to the blocks.[7] Contrary to this, Buckenmaier III *et al*., reported lumbar plexus continuous peripheral nerve block (CPNB) in patients on LMWH therapy without any catheter-related bleeding complications.[8] However, the authors agree that the study population included young and otherwise healthy trauma patients and were of insufficient number to conclude safety of the procedure. Similarly, a retrospective analysis also reported safety of peripheral blocks both for administration and removal of catheter, irrespective of the type of anticoagulants and the dose regimen, but the study did not mention about close postoperative monitoring for bleeding complications.[9]

American Society of Regional Anaesthesia and Pain Medicine (ASRA)[10] has defined guidelines for placement and removal of catheter for continuous plexus blocks or for deep blocks in anticoagulated patients. They advice the same guidelines to be applied on PNBs admitting that, if applied, the restrictions may become more than necessary.[10]

In clinical practice, the risk benefit assessment of a nerve block needs to be evaluated depending on the urgency of the surgical procedure. Thus, one may be justified in attempting regional blocks despite being on anticoagulant therapy especially in potentially life-threatening limb salvage situations. Unlike CNB, in PNB, the haematoma is easier to detect both clinically as well as radiologically (ultrasonogram) owing to relative superficial level of nerves. Nevertheless, close monitoring of limb for sensory and motor recovery is required. Persistent pain at the site, drop in haemoglobin levels, skin morphologic changes and neurological deficits may indicate underlying haematoma. In cases of any doubt, more frequent ultrasonograms at regular intervals, and rarely a computerized tomography (CT) scan, may be useful to rule out the compressing haematoma on the nerve. In the case of expanding haematoma, reversal of anticoagulation should be considered. Thus, when facilities exist, an ultrasonogram can be used both for performing nerve blocks as well as postoperatively to improve the safety and accuracy.
